# Bacteriolytic Activity Of Human Interleukin-2, Chicken Egg Lysozyme In The Presence Of Potential Effectors

**Published:** 2017

**Authors:** P. A. Levashov, D. A. Matolygina, E. D. Ovchinnikova, D. L. Atroshenko, S. S. Savin, N. G. Belogurova, S. A. Smirnov, V. I. Tishkov, A. V. Levashov

**Affiliations:** Department of Chemical Enzymology, Faculty of Chemistry, M.V. Lomonosov Moscow State University, Leninskie Gory, 1–3, Moscow, 119991, Russia; Bach Institute of Biochemistry, Research Center of Biotechnology of the Russian Academy of Sciences, Leninsky Ave. 33, bldg. 2, Moscow, 119071, Russia

**Keywords:** bacteriolytic activity, chicken egg lysozyme, interleukin-2

## Abstract

The bacteriolytic activity of interleukin-2 and chicken egg lysozyme in the presence of various substances has been studied. Glycine and lysine do not affect the activity of interleukin-2 but increase that of lysozyme, showing a bell-shape concentration dependence peaking at 1.5 mM glycine and 18 mM lysine. Arginine and glutamate activate both interleukin-2 and lysozyme with a concentration dependence of the saturation type. Aromatic amino acids have almost no effect on the activity of both interleukin-2 and lysozyme. Aromatic amines, tryptamine, and tyramine activate interleukin-2 but inhibit lysozyme. Peptide antibiotics affect interleukin and lysozyme similarly and exhibit maximum activity in the micromolar range of antibiotics. Taurine has no effect on the activity of interleukin-2 and lysozyme. Mildronate showed no influence on lysozyme, but it activated interleukin-2 with the activity maximum at 3 mM. EDTA activates both interleukin-2 and lysozyme at concentrations above 0.15 mM.

## INTRODUCTION

Interleukin-2 plays a key role in the regulation of the immune system and is used as medication for various oncological diseases [1, 2]. This cytokine was recently shown to exhibit bacteriolytic acitivity [3-6]. The physiological significance of the recently identified bacteriolytic activity for this important cytokine is unclear. Interleukin-2 shows a substrate specificity distinct from that of chicken egg lysozyme [3-6]. However, there are microorganisms that are affected by both interleukin-2 and lysozyme. This work has aimed at identifying the potential effectors of interleukin and lysozyme activity by a direct comparison under identical experimental conditions. A series of amino acids of various types, biogenic amines, peptide antibiotics, EDTA, and mildronate were selected as model compounds, since biological systems may contain these compounds or their analogs. *Escherichia coli *cells were taken as the model substrate, because they undergo lysis with both interleukin-2 and lysozyme [3-5]. This study on the character of the effect of various additives may help in future elucidation of the mechanism of interleukin-2 bacteriolytic activity. In addition, an understanding of the peculiarities of the effects of various compounds on interleukin-2 and lysozyme activity may provide a clue in future efforts directed towards enhancing the efficiency of existing medication, as well as designing new ones.

## EXPERIMENTAL

The following materials were used: glycine (Fluka, Germany); EDTA (Panreac, Spain); L-lysine (Serva, Germany); tyramine, triptamine, taurine (Acros Organics, USA), Tris, MES (Amresco, USA); bacitracin (MP Biomedicals, Germany); polymyxin B, L-tryptophane, L-tyrosine, L-phenylalanine, chicken egg lysozyme (Sigma-Aldrich, USA); NaOH (Merck, Germany); acetic acid (ChemMed, Russia); hydrochloric acid (Laverna, Russia); mildronate (2- (2-carboxylatoethyl)-1,1,1-trimethylhydrazinium) (Cridex, Latvia); sodium L-glutamate (HongMei, China); Roncoleukin®, the 0.25 mg/mL solution of purified recombinant interleukin-2 for intravenous and subcutaneous injections (Biotech, Russia).

The *E. coli *JM109 strain used in this work was provided by Dr. J.Messing (Waksman Institute, New Jersey, USA). The cells were grown in accordance with the standard protocol [7]. The 109 CFU/mL cell sus pension in 0.15 M NaCl was frozen by immersing 1 mL aliquots into liquid nitrogen. The cells were stored at –70°C for no longer than for 2–3 weeks. The cells were thawed right before the experiment. The thawed cell suspension was centrifuged at 4,500 rpm for 5 min in a Minispin centrifuge (Eppendorf, Germany) and then re-suspended in the assay buffer.

Bacteriolytic activity (as the rate of cell lysis) was measured turbidimetrically by following the decrease in the suspension absorbance, –dA/dt, min–1 [5, 8] at 650 nm, which is linearly dependent on the rate of cell count changes, dCFU/dt, under these conditions. The measurements were taken in a cuvette with a 1-cm light path and 0.5mL volume; the absorbance was measured on a UV-1800 spectrophotometer (Shimadzu, Japan). A lysozyme solution was prepared right before the experiment by dissolving in the assay buffer. The commercial solution of interleukin-2 was used without additional purification, and an ampoule was opened just before the experiment. The bacteriolytic activity measurements were assayed at 37°C in a 10 mM MES-Tris-acetate buffer, pH 8.8 for interleukin-2, and pH 8.5 for lysozyme. The final concentrations of interleukin-2 and lysozyme were equal to 15 μg/mL and 0.1 μg/mL, respectively, to ensure comparable values of cell lysis rates. The cell suspension was mixed with the buffer in the cuvette to achieve an initial absorbance (A650) of 0.43–0.45. The background changes in the absorbance were recorded for 5 min to account for the cell’s self-lysis or precipitation. Then, the effectors under study were added and the background absorbance changes recorded for 5 min; this was followed by the addition of the enzyme. The initial rate of cell lysis was determined from the absorbance changes in a timeframe from 5–25 s after enzyme addition. The background rate for cell self-lysis or precipitation was subtracted from the initial rate of cell lysis in the presence of the enzyme. In all experiments, the background rate value did not exceed the average value of a standard deviation for the cell lysis rates determined in the enzyme presence. All added compounds (except for the enzyme) did not change the background lysis rates within the experimental error. The pH value for the compounds under study was tested before the addition and adjusted to 8.8 (8.5) with NaOH or HCl solutions if necessary. The effects of the additives observed in this experiment did not originate from the activity changes caused by the changes in the ionic strength: within the range of ionic strength changes in this work, no significant changes in the bacteriolytic activity were observed [3, 8].

## RESULTS AND DISCUSSION

**Fig. 1:**
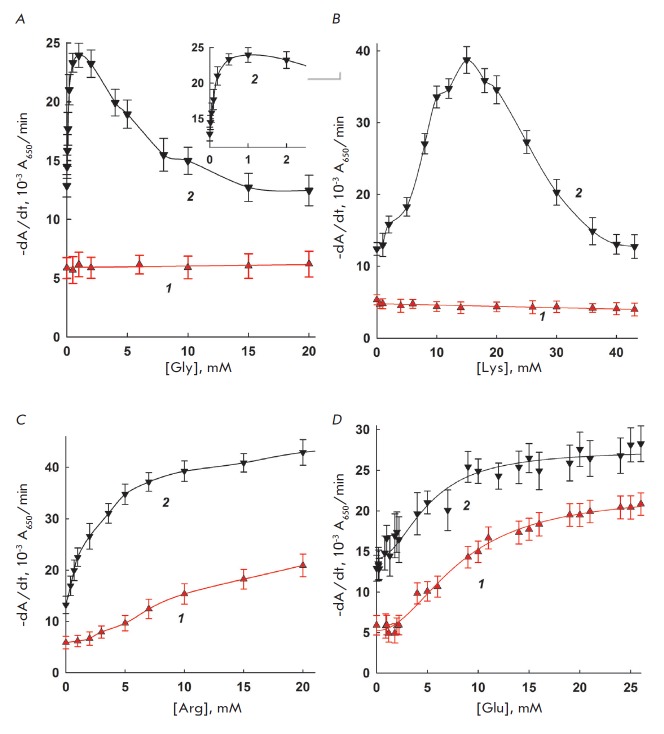
The dependence of interleukin-2 (1) and lysozyme (2) activity on the concentration of added glycine (1A), lysine (1B), arginine (1C), and glutamate (1D). 37°C, 10 mM MES-Tris-acetate buffer, pH 8.8, and pH 8.5 for interleukin-2 and lysozyme, respectively.

The dependences of interleukin-2 and lysozyme activity on the concentration of glycine, lysine, arginine, and glutamate are shown in *Fig. 1*. As seen in *Fig. 1 A and B*, the activity of interleukin-2 in the presence of glycine, the simplest based on structure natural amino acid, and positively charged lysine remained unchanged. For lysozyme, a maximum was observed at 2 mM glycine or 15–18 mM lysine, where lysozyme activity was significantly higher than the original. A further increase in the concentrations of glycine and lysine returned the lysozyme activity to its original level. Hence, lysozyme and interleukin-2 show completely different behaviors in the presence of these two amino acids, and this may point to the difference in their mechanisms of action. Such effect of lysozyme activity enhancement in the presence of glycine has never been reported in the literature. However, it is known that glycine, in addition to its bacteriostatic properties, may increase the efficiency of various antimicrobial agents [9]. The distinct action of glycine on lysozyme and interleukin-2 is difficult to explain. One may speculate that glycine affects one of the bacterial-type porines to ease the lysozyme interaction with the cell wall, and that at the same time it has no effect on the action of interleukin-2.

The effect of arginine on lysozyme and interleukin-2 bacteriolytic activity is shown in *Fig. 1C*. As seen, in both cases, a significant increase in cell lysis rates is observed at effector concentrations of 10 mM and higher. The activation by arginine could be of a complex nature and reflect a combination of arginine effects on the enzyme and the cell: it is well-known that arginine enhances the efficiency of lysozyme-based pharmaceuticals by diminishing protein aggregation [10]. It is also necessary to mention that the dependences of arginine and lysine on the bacteriolytic activity show stark differences. Probably, this difference is due to the various polarities and geometries of positively charged side chains.

A similar trend in the changes in lysozyme and interleukin-2 activity is observed in the presence of glutamate: a 2-fold increase for lysozyme and 3-fold increase for interleukin-2 at 15 mM glutamate. Further increase in the glutamate concentrations does not significantly change this activity, which approaches a manner of threshold. A similar effect by glutamate on the activity of lysozyme and interleukin-2 can be explained based on the hypothesis that glutamate forms a complex with positively charged groups on the protein surface, preventing various types of nonproductive enzyme adsorption on cells, which may significantly change the apparent values of bacteriolytic activity parameters [11, 12].

**Fig. 2:**
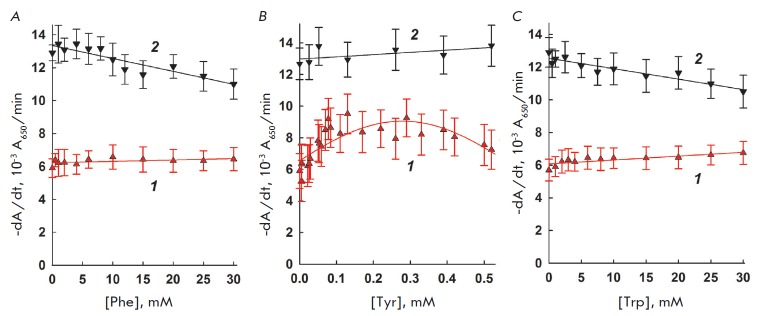
The dependence of interleukin-2 (1) and lysozyme (2) activity on the concentration of added phenylalanine (2A), tyrosine (2B), and tryptophan (2C). 37°C, 10 mM MES-Tris-acetate buffer, pH 8.8, and pH 8.5 for interleukin-2 and lysozyme, respectively.

The dependence of lysozyme and interleukin-2 activity on the concentration of aromatic amino acids is shown in *Fig. 2*. For tyrosine, the highest concentration used was restricted to 0.6 mM because of its low solubil ity in water. As seen, in the presence of phenylalanine and tryptophan, the small reduction in lysozyme activity is negligible within the experimental error. The apparent increase in lysozyme activity in the presence of tyrosine is also within the experimental error. Interleukin-2 activity in the presence of phenylalanine and tryptophan is unchanged. The dependence of interleukin-2 activity on the tyrosine concentration shows a 30% increase at 0.25–0.3 mM. The general conclusion is that aromatic amino acids have no significant effect on the activity of lysozyme, as well as on interleukin-2. A completely different picture emerges for aromatic amino acid derivatives: namely, biogenic aromatic amines – tryptamine and tyramine – as discussed below.

**Fig. 3:**
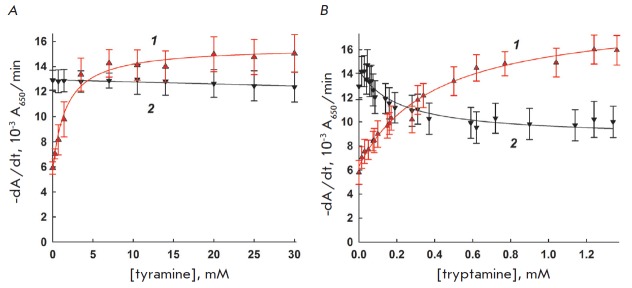
The dependence of interleukin-2 (1) and lysozyme (2) activity on the concentration of added tyramine (3A) and tryptamine (3B). 37°C, 10 mM MES-Tris-acetate buffer, pH 8.8, and pH 8.5 for interleukin-2 and lysozyme, respectively.

The dependence of interleukin-2 and lysozyme activity on the concentrations of the biogenic amines tyramine and tryptamine, which can be formally considered as derivatives of the tyrosine and tryptophan amino acids, is shown in *Fig. 3*. As can be seen, interleukin-2 is activated by either biogenic amine, whereas the activity of lysozyme is inhibited. This result may be used as proof of the substantive differences between interleukin-2 and lysozyme with respect to their mechanism of action. Interleukin-2 is prone to binding to various ligands via hydrophobic interactions [13]: hence, it is possible that tyramine and tryptamine bind to some hydrophobic loci on the interleukin-2 surface, lowering its nonproductive adsorption on cells.

**Fig. 4:**
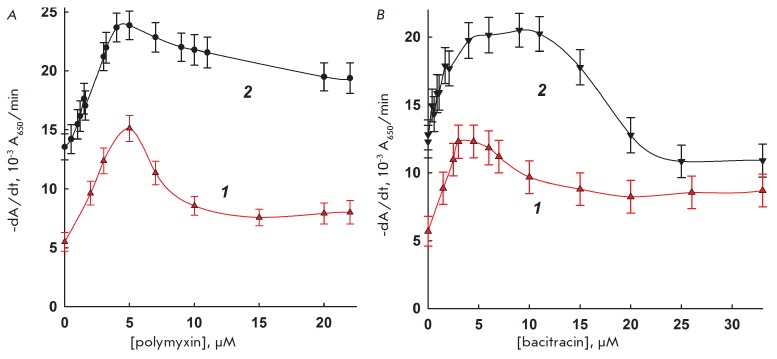
The dependence of interleukin-2 (1) and lysozyme (2) activity on the concentration of added polymyxin B (4A) and bacitracin (4B). 37°C, 10 mM MES-Tris-acetate buffer, pH 8.8, and pH 8.5 for interleukin-2 and lysozyme, respectively.

The dependence of interleukin-2 and lysozyme activity on the concentrations of the peptide antibiotics polymyxin B and bacitracin is shown in *Fig. 4*. A similar picture is observed for both bacteriolytic factors and both antibiotics: an activity maximum at 5–7 μM. These peptide antibiotics are known cytostatics for *E.coli *[14, 15]: hence, the similarity in the observed effects may originate from their direct action on the cells and not from a modulation of the properties of bacteriolytic factors. The antibiotic by itself cannot cause cell lysis but renders a cell more sensitive to bacteriolytic enzymes, as was observed for endolysine from bacteriohages [16].

**Fig. 5:**
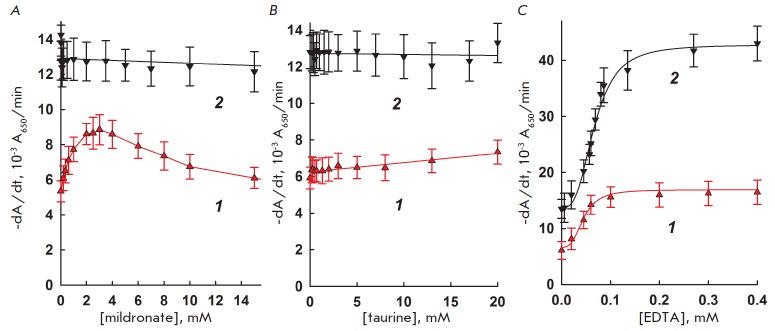
The dependence of interleukin-2 (1) and lysozyme (2) activity on the concentration of added mildronate (5A), taurine (5B), and EDTA (5C). 37°C, 10 mM MES-Tris-acetate buffer, pH 8.8, and pH 8.5 for interleukin-2 and lysozyme, respectively.

The dependence of interleukin-2 and lysozyme activity on the concentrations of mildronate, taurine, and EDTA is shown in *Fig. 5*. Mildronate has no effect on the activity of lysozyme but increases the activity of interleukin-2: the maximum is observed at 3 mM. The physiological effects of mildronate are usually explained by its similarity to natural, biologically active compounds, and γ-butyrobetaine in particular or its derivatives: for example, L-carnitine [17, 18]. Mildronate binds to and inhibits γ-butyrobetaine hydroxylase (IC_50_ = 62 μM) and carnitine acetyltransferase (IC_50_ = 1.6 mM). So, it may also bind other proteins and change their conformation and properties. Taurine has no effect on the activity of interleukin-2 and lysozyme. EDTA at concentrations above 0.1 mM enhances the effect of both bacteriolytic factors, and similarly to peptide antibiotics, its effect, at least in part, can be explained by the effect of EDTA on cells, and not on the enzyme.

## CONCLUSION

Thus, the effect of additives on interleukin-2 and lysozyme depends on the chemical nature of the additives. This can be indicative of different mechanisms of action. We have identified substances which activate these bacteriolytic factors. This can be of practical importance. Effectors can be used to improve the effectiveness of existing medication, as well as to create new medicinal compositions. For example, our research shows that glycine, lysine, and glutamate enhance the bacteriolytic activity of lysozyme. Glycine, lysine, and lysozyme are widely used as drugs, but their combined action has not been studied. The effect of glutamate and arginine on the activity of lysozyme had also not been investigated previously. In current medical practice, interleukin-2 is used as a regulator of the immune system but not as a bacteriolytic factor, since its bacteriolytic properties had not been previously known. However, it is possible that antimicrobial properties also play an important role in some cases when the effectiveness of interleukin-2 is confirmed. Interleukin-2 is used both in the case of sepsis, where the role of bacteria is obvious, and in the treatment of cancer, where the role of bacteria is less obvious but there may be a combination of bacterial tissue damage and the underlying disease. The mechanism of bacteriolytic action of interleukin-2 has not yet been established, and the mechanism of action of effectors on interleukin-2 activity also requires further investigation. It has become clear that special attention should be focused on the activation of interleukin-2 in the presence of additives: for example, mildronate, arginine, and glutamate. Combined use of these drugs could open new possibilities in the treatment of serious diseases.
